# Transcutaneous electric nerve stimulation over acupoints for patients with diarrhea-predominant irritable bowel syndrome: Protocol for systematic review and meta-analysis

**DOI:** 10.1097/MD.0000000000013267

**Published:** 2018-12-21

**Authors:** Bo-yu Han, Qian-Feng Shao, Yu Cong, Song Guo, Xin-Yong Mao, Ru-Han Wei, Wei Wei

**Affiliations:** a Dongzhimen Hospital, Beijing University of Chinese Medicine; b Beijing Key Laboratory of Functional Gastrointestinal Disorders Diagnosis and Treatment of Traditional Chinese Medicine, Wangjing Hospital, China Academy of Chinese Medical Sciences, Beijing, China; c Cleveland State University, Cleveland, OH.

**Keywords:** irritable bowel syndrome with diarrhea, meta-analysis, protocol, systematic review, transcutaneous electric nerve stimulation over acupoints

## Abstract

**Background:**

At present, drug therapy for diarrhea-predominant irritable bowel syndrome (IBS-D) has made great progress; however, it does not often produce a satisfying curative effect. Transcutaneous electric nerve stimulation over acupoints (Acu-TENS) might be more effective in improving patient's symptoms and producing fewer side-effects as a result.

Although with a great progress of the drug therapy for IBS-D, it is often hard to achieve its satisfactory curative effect. Acu-TENS that may be effective to improve patients’ symptoms and fewer side-effects will be sought. There is no systematic review concerning the efficacy of Acu-TENS for IBS-D published. Therefore, this review aims to systematically evaluate the efficacy of Acu-TENS on IBS-D.

**Methods:**

Four English (PubMed, EMBASE, The Cochrane Library, Web of Science) and 4 Chinese electronic databases (Biomedical Literature Database, CNKI, VIP, Wanfang Database) will be searched from their inception to November 26, 2018. Randomized controlled trials that evaluated the effect of Acu-TENS on patients with IBS-D will be included. The primary outcome measures will include average weekly stool frequency, visual analog scale (VAS), and the Bristol scale. The secondary outcome measures will include the MOS 36-item short-form health survey (SF-36), IBS Quality of Life Questionnaire (IBS-QOL), severity of IBS symptoms (IBS-SSS), and rectal perception. Quality evaluation and data extraction will be independently undertaken, respectively. The data from the eligible trials will be analyzed by RevMan5.3.

**Results:**

For patients with IBS-D, this systematic review will provide evidences related to the efficacy of Acu-TENS in these evaluation aspects, stool frequency, VAS and the Bristol scale, SF-36, IBS-QOL, IBS-SSS, and rectal perception.

**Conclusion:**

This evidence may be useful to medical workers with regard to the use of Acu-TENS in the treatment of IBS-D.

**PROSPERO registration number:** PROSPERO CRD442018109294.

## 1 Introduction

Irritable bowel syndrome (IBS) is a common chronic functional gastrointestinal disorder characterized by recurrent abdominal pain and/or bloating related to defecation without reliable biological markers.^[1]^ The prevalence of IBS in Asia using Rome criteria was approximately 4.6% to 21.2% in adults, global prevalence of IBS was demonstrated in a meta-analysis of 11%.^[2–4]^ IBS is associated with substantial burden, such as higher levels of anxiety, lost productivity at work, work absenteeism.^[5]^ IBS-D is the common subtype, which accounts for 23.4% to 40% of all IBS patients.^[4]^ IBS-D poses a substantial economic burden on the global healthcare system. Patients with IBS-D compared with the unaffected controls had significantly higher total all-cause healthcare costs ($9436 vs $7169; *P* < .001).^[6]^ Although with a great progress of the drug therapy for IBS-D which have been proven to be effective in relieving symptoms and improving quality of life for patients with IBS-D, it is often hard to achieve the satisfactory curative effect. These drugs include antidepressants, antibiotics, probiotics, and serotonin receptor modulators.^[7,8]^ The temporary and limited effect remains to be a difficult problem on account of the mechanisms by which symptoms that arise are poorly understood.^[9]^ Owing to limited effect and the side effects of medications, patients with IBS-D often cannot get satisfying curative effect.^[10]^ Therefore, an increasing number of patients tend to use complementary and alternative therapy.^[11–13]^

As an alternative therapy, transcutaneous electric nerve stimulation (TENS) has been increasingly studied in clinical practice, a Western treatment acts on the afferent nerve fibers to stimulate the nerves for therapeutic purposes.^[14]^ TENS over acupoints (Acu-TENS) is a coordinated intervention merging TENS with acupuncture. Compared with traditional acupuncture and electro-acupuncture, it is not necessary to insert a needle into acupoints for stimulation. In recent years, it has been widely used in clinical practice.^[15–17]^

Considering there is a limited evidence concerning its efficacy for IBS-D, we performed this review that aims to systematically evaluate the efficacy of Acu-TENS for IBS-D and thus to provide a reliable evidence for clinical decision.

## 2 Methods and analysis

### 2.1 Inclusion criteria for study selection

All randomized controlled trials evaluating the effect of Acu-TENS comparing no interventions, placebo control, sham Acu-TENS on IBS-D will be included. Participants who are diagnosed with IBS-D according to the Rome II, III, or VI criteria will be included, regardless of their age, gender, and ethnicity. We will exclude those who had an acute exacerbation within 1 week before the study.

### 2.2 Outcome measures

Primary outcome measures will include average weekly stool frequency, visual analog scale (VAS), and the Bristol scale.^[13]^ Secondary outcome measures will include SF-36, IBS-QOL, IBS-SSS, and rectal perception.^[18–21]^ Rectal sensory thresholds will be evaluated by rectal balloon distension.

### 2.3 Literature search

Four English (PubMed, Embase, The Cochrane Library, Web of Science) and 4 Chinese electronic databases (Biomedical Literature Database, CNKI, VIP, and Wanfang Database) will be searched from their inception to November 26, 2018. There was no limit to the type of language. Reference lists of eligible studies will be reviewed to discover further eligible studies. We have drawn up detailed search strategies for each electronic database to identify eligible studies totally. The search strategy is shown in Table 1.

**Table 1:**
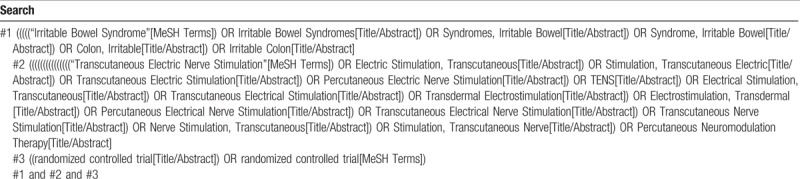
Search strategy.

### 2.4 Study selection

**Figure 1:**
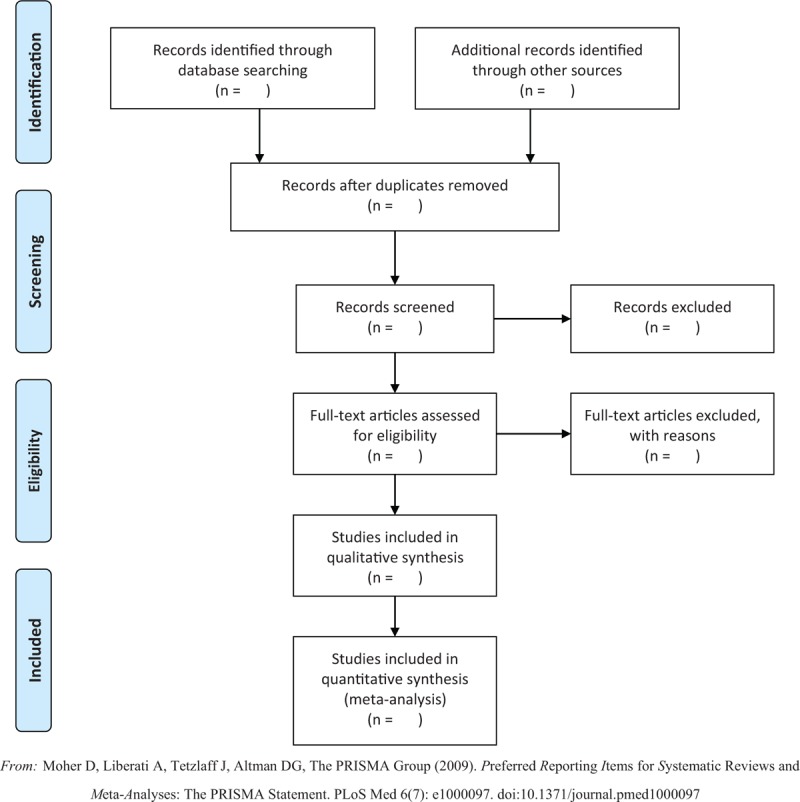
Study flow diagram. *From:* Moher D, Liberati A, Tetzlaff J, Altman DG, The PRISMA Group (2009). *P*referred *R*eporting *I*tems for *S*ystematic Reviews and *M*eta-*A*nalyses: The PRISMA Statement. PLoS Med 6 (7): e1000097. doi:10.1371/journal.pmed1000097.

Two review authors (B-YH, Q-FS) will screen and extract independently titles and abstracts, then select potentially eligible studies. Finally, the full-text of literature will be reviewed carefully according to the inclusion and exclusion criteria. Disagreements and inconsistency will be resolved by a third review author (YC). The study flow diagram is shown in Fig. 1.

### 2.5 Data extraction

Two researchers will independently extract the data, the following content will be included: first author, the year of publishing, diagnosis criteria, study population, treatment protocol, outcome measurements, duration of treatment, duration of the follow-up period, and baseline characteristic. A third review author will resolve divergences through discussion.

### 2.6 Risk of bias assessment

The Cochrane risk of bias tool will be used to evaluate methodologic quality, which is described in the Cochrane Handbook of Systematic Reviews of Interventions:

- Sequence generation (selection bias)
- Allocation sequence concealment (selection bias)
- Blinding of participants and personnel (performance bias)
- Blinding of outcome assessment (detection bias)
- Incomplete outcome data (attrition bias)
- Selective outcome reporting (reporting bias) and other potential sources of bias

### 2.7 Statistical analysis

The data of all the eligible trials will be analyzed by RevMan5.3. Continuous data will be calculated by the mean differences and 95% confidence interval (95% CI), and dichotomous data will be calculated by the relative risks (RRs) and 95% CI. Heterogeneity will be assessed by the *I*-squared (*I*^2^) statistic. We will regard as substantial heterogeneity when *I*^2^ > 50% or *P* < .05, and a random effect model will be chosen. Otherwise, a fixed effects model will be applied to calculate the pooled RR. We will conduct subgroup analysis and sensitivity analysis if necessary.

### 2.8 Grading the quality of evidence

The Grading of Recommendations Assessment, Development, and Evaluation will be used to assess the quality of evidence for the outcomes.^[22]^ The quality of outcome measures will be categorized into 4 levels: high, moderate, low, and very low quality.

## 3 Discussion

Owing to less high-level evidence-based medical research evaluating the efficacy of Acu-TENS on IBS-D, Acu-TENS has been gradually accepted and widely used in the treatment of IBS-D. To our knowledge, this is the first systematic review to investigate the efficacy of Acu-TENS for IBS-D. This review will provide evidence related to the efficacy of Acu-TENS in these evaluation aspects, stool frequency, VAS and the Bristol scale, SF-36, IBS-QOL, IBS-SSS, and rectal perception. It may be useful to medical workers considering the use of Acu-TENS in the treatment of IBS-D.

## Author contributions

Conceptualization: Bo-yu Han, Wei Wei

Data curation: Yu Cong, Song Guo

Formal analysis: Qian-Feng Shao, Xin-Yong Mao

Writing – original draft: Bo-yu Han

Writing – review & editing: Ru-Han Wei, Wei Wei

**Data curation:** Yu Cong, Song Guo.

**Formal analysis:** Qian-Feng Shao, Xin-Yong Mao.

**Writing – original draft:** Bo-yu Han.

**Writing – review & editing:** Ru-Han Wei, Wei Wei.
